# Nutrigenomics and Breast Cancer: State-of-Art, Future Perspectives and Insights for Prevention

**DOI:** 10.3390/nu12020512

**Published:** 2020-02-18

**Authors:** Maha Sellami, Nicola Luigi Bragazzi

**Affiliations:** 1 Sport Science Program (SSP), College of Arts and Sciences (CAS), Qatar University, Doha 2713, Qatar; 2 Postgraduate School of Public Health, Department of Health Sciences (DISSAL), University if Genoa, 16132 Genoa, Italy; 3 Laboratory for Industrial and Applied Mathematics (LIAM), Department of Mathematics and Statistics, York University, Toronto, ON M3J 1P3, Canada

**Keywords:** breast cancer, preventive medicine, nutrigenomics, nutriproteomics

## Abstract

Proper nutrition plays a major role in preventing diseases and, therefore, nutritional interventions constitute crucial strategies in the field of Public Health. Nutrigenomics and nutriproteomics are arising from the integration of nutritional, genomics and proteomics specialties in the era of postgenomics medicine. In particular, nutrigenomics and nutriproteomics focus on the interaction between nutrients and the human genome and proteome, respectively, providing insights into the role of diet in carcinogenesis. Further omics disciplines, like metabonomics, interactomics and microbiomics, are expected to provide a better understanding of nutrition and its underlying factors. These fields represent an unprecedented opportunity for the development of personalized diets in women at risk of developing breast cancer.

## 1. Breast Cancer

Despite important research advancements and clinical improvements, breast cancer still represents a serious Public Health challenge, in terms of both epidemiological and economic burden [1]. Genetics and genomics have contributed to revolutionizing the diagnostic approach as well as the prognosis and outcomes of breast tumor, paving the way for personalized and tailored ad hoc treatments, which are currently under experimentation [2]. Breast tumor is still, however, a leading cause of mortality [1], with an increased incidence up to 1,960,681 cases, contributing to 17,708,600 disability-adjusted life years (DALYs) in 2017 [3].

Even though common sense seems to suggest that nutritional factors can have a key role in breast tumor aetiopathogenesis and prevention, the application of methods based on rigorous approaches, such as systematic reviews and meta-analyses, has led to mixed results [4].

It has been hypothesized that classical surveys, relying upon nutritional epidemiology tools and instruments, are plagued by errors and biases, such as inadequate nutritional assessment, population sampling or lack of a proper follow-up [4]. Further, these studies tend to consider breast cancer as a “one-size-fits-it-all” disease, not being able to capture the high degree of heterogeneity of breast tumors. The real picture is more complex, in that breast cancer is heterogeneous from a histological point of view (ductal and lobular, which can be, in turn, further subdivided into other categories, among others) and, above all, from a molecular standpoint (Luminal A and B, triple negative/basal-like, and HER-2 type) [5,6].

Nutrigenomics is emerging as a new specialty from the intersection of genomics and nutritional disciplines. It is anticipated to play a fundamental role in breast tumor prevention and early detection, in that the “identification of the relationship between nutrition and breast cancer among sporadic cases and gene mutation carriers provides necessary data for breast cancer prevention” [7].

The purpose of the present overview is to provide an updated synthesis of the current knowledge of the effects of nutrients and diet on breast tumor, from a nutrigenomic perspective. A narrative synthesis of the major studies on the topic was undertaken, by searching in PubMed/MEDLINE. Keywords included some major nutrients, breast cancer and breast cancer-related genes.

## 2. The Traditional Framework: Nutrition and Breast Cancer

“Concerning diet, only alcohol is widely recognized for being most consistently associated with breast cancer risk. Diet seems to be modestly associated with the disease, highlighting the need for more studies to be conducted” [8].

Adherence to a Mediterranean diet does not seem to reduce breast-cancer-related specific risk in terms of incidence rate and mortality according to a systematic review of the literature and a meta-analysis of observational studies [9], whilst another recent meta-analysis reaches opposing conclusions [10], reporting, instead, a protective role of Mediterranean diet (risk-ratio or RR 0.93 (95% confidence interval or CI 0.87–0.99)). This conflict may be due, besides the above-mentioned shortcomings, also to methodological issues, such as the choice of studies to be included, different definitions of Mediterranean diet [11], as well as to the study design, with cohort studies giving more contrasting findings than case-control studies [12].

Concerning saturated fat intake, breast-cancer-specific death (hazard-ratio or HR 1.63 (95% CI 1.19–2.24)) is higher for women consuming high amounts of fats [13]. Cholesterol uptake from diet is associated with an increased breast cancer risk (1.29 (95% CI 1.06–1.56)) [14].

Concerning meat, based on the findings of a meta-analysis of prospective studies, RR of breast cancer for the highest versus the lowest consumption categories resulted in 1.10 (95% CI 1.02–1.19) for red meat, and 1.08 (95% CI 1.01–1.15) for processed meat. In detail, RR was 1.11 (95% CI 1.05, 1.16) for a daily increased uptake of 120 g of red meat, and 1.09 (95% CI 1.03, 1.16) for a daily increased consumption of 50 g of processed meat [15]. A previous meta-analysis of cohort studies reached opposite conclusions [16]. The authors stated that a study based on a molecular classification according to the hormone receptor status may have been more accurate. However, a previous meta-analysis pooling together case-control and cohort studies was able to find a positive correlation between meat intake and risk of developing breast cancer (RR 1.17 (95% CI 1.06–1.29)) [17].

Regarding dairy intake, a recent meta-analysis found that a high and modest consumption (greater than 600 g/day and in the range 400–600 g/day, respectively) were able to reduce the risk of breast cancer compared with low dairy intake (less than 400 g/day; RR 0.90 (95% CI 0.83–0.98), and RR 0.94 (95% CI 0.91–0.98), respectively). The dose–response analysis showed a linear relationship between dairy consumption and breast cancer risk. In particular, subgroup analysis showed that yogurt (RR 0.91 (95% CI 0.83–0.99)) and low-fat dairy product types (RR 0.85 (95% CI 0.75–0.96)) decreased the risk of cancer, while other dairy products failed to exert such effects [18].

Consuming five or more than five eggs weekly correlated with an increased risk of developing breast cancer (RR 1.04 (95% CI 1.01–1.07) for consuming five eggs per week and 1.09 (95% CI 1.03–1.15) for consuming around nine eggs/week) [19]. A previous meta-analysis found similar results, but not for case-control studies, American and pre-menopausal subjects, as well as for those consuming > 5 eggs/week [20].

Prospective epidemiological investigations showed a U-shaped association between dietary folate uptake and the risk of developing breast cancer. In detail, women with a dietary folate intake ranging from 153 to 400 μg/day showed a significant reduced breast cancer risk compared with those consuming less than 153 μg/day, but not for those consuming more than 400 μg per day [21].

When comparing the highest versus the lowest quartiles of intake, weak, non-statistically significant relationships could be reported for fruits and vegetables. No further healthy advantage emerged comparing the highest and lowest uptake deciles. No correlations could be detected for specific vegetables or fruits [22].

Concerning some specific fruits, apple was found to exert a protective effect only in case-control studies (odds-ratio or OR 0.79 (95% CI 0.73–0.87)), but not in prospective studies [23]. Grapefruit, which inhibits cytochrome P450 3A4 and may affect estrogen metabolism, was not found to have any effect on breast cancer risk [24]. Citrus fruit, on the contrary, seemed to have a protective effect (OR 0.90 (95% CI 0.85–0.96)), according to a meta-analysis of case-control studies [25]. Somasundaram and collaborators demonstrated that a molecule termed limonin, contained in this fruit, may be beneficial for patients undergoing chemotherapy [26].

A vegetarian diet did not confer advantages with respect to a non-vegetarian diet [27]. However, a meta-analysis reported a protective role of consumption of cruciferous vegetables (RR 0.85 (95% CI 0.77–0.94)) [28].

The uptake of soy, an important source of phytoestrogens and isoflavones, like daidzein (4′,7-dihydroxyisoflavone) and genistein (4′,5,7-trihydroxyisoflavone), was found to significantly correlate with a decreased risk of developing breast cancer (OR 0.78). This could be due to: (1) increased differentiation of the mammary tissue, (2) reduced bio-activation and bio-transformation from pro-carcinogens to carcinogens and (3) modulation of genes belonging to signal transduction pathways and cascades underlying tumor initiation, promotion and/or progression, such as the BRCA1/BRCA2 pathway [29].

Ethnic factor is another parameter that could explain discrepancies in the literature. For instance, another meta-analysis showed protective effect of soy only for Asian women, both for pre- (OR 0.59 (95% CI 0.48–0.69)) and post-menopausal (OR 0.59 (95% CI 0.44–0.74)) subjects, but not for Western individuals [30].

Concerning fish intake, a meta-analysis of prospective cohort studies found that marine n-3 polyunsaturated fatty acids assumption determined a 14% reduction in breast cancer risk [31,32]. Alpha linolenic acid, on the contrary, was not associated with breast cancer risk [31]. Omega-3/omega-6 lipids consumption can lead to a decreased risk of developing breast cancer [33,34]. 

Alcohol uptake correlates with an increased risk of having highly dense glands [35] and developing breast cancer (RR 1.61) [36]. Alcohol-related risk of inducing breast cancer may depend also on the menopausal status of women [37].

Alcohol consumption after receiving a diagnosis of breast cancer did not correlate with overall survival, whereas better survival was reported in cases of pre-diagnosis consumption. Taking into account the estrogen receptor (ER) status, pre- or post-diagnosis alcohol consumption was not associated with breast-cancer-specific mortality in ER-positive breast cancer women. Moderate post-diagnosis alcohol consumption correlated with a small decrease in breast-cancer-specific mortality rate in women with ER-negative breast cancer. In conclusion, alcohol consumption after a diagnosis of breast tumor does not seem to be associated with health outcome and prognosis [38].

Coffee uptake does not seem to be correlated with the risk of developing breast cancer [39], even though a previous meta-analysis gave contrasting findings [40]. A recent meta-analysis found, instead, a weak association when focusing on post-menopausal women and BRCA1 mutation carriers [41]. Li and collaborators, instead, found a statistically significant, inverse relationship between caffeine uptake and breast cancer risk in ER-negative women [42].

Finally, tea consumption has not been generally found to correlate with breast cancer risk, apart from drinking black tea, which may be associated with an increased risk [43]. However, a previous meta-analysis failed to find any association between black tea and breast tumor [44].

The effects of major nutrients in terms of breast cancer risk are summarized in Table 1.

## 3. The New Framework: Nutrition, Genes and Breast Cancer

The previously reported discrepancies can be explained, taking into account several parameters: methodological aspects (case-control study, prospective, cross-sectional study, etc.), the characteristics of the samples recruited and, above all, the molecular phenotype/endo-phenotype [45]. In the next paragraph, we will overview the main interactions between genes associated with breast cancer and nutrients.

### 3.1. BRCA1, Diet and Breast Cancer

The “Breast Cancer Type 1 susceptibility gene” (BRCA1, 17q21.31) is a tumor suppressor that acts as a caretaker gene, that is to say, it is responsible for repairing DNA lesions and mutations, such as double-strand breaks, together with other oncosuppressors [46,47]. The protein exerts various biological functions, including cell cycle, transcription, genomic stability, ubiquitination, and transcriptional regulation [48].

As previously mentioned, inconsistency among studies assessing coffee uptake and breast cancer risk could be solved, taking into account BRCA1 mutations. Nikitina and collaborators found that, in BRCA1 mutation carriers, coffee uptake correlated with lower levels of micronuclei, demonstrating its preventive effect, potentially mediated by increased biochemical activities to sense and repair DNA damages [49].

Utilizing a murine and an in-vitro cellular model, Donovan and colleagues [50] assessed the effect of a genistein-enriched (4 and 10 ppm) diet. Authors found that genistein supplementation resulted in up-regulation of BRCA1 protein levels, in reduced levels of CpG methylation, and in a decreased aryl hydrocarbon receptor (AHR)-binding affinity at the level of BRCA1 exon 1a. Similar findings were obtained by Cabanes and colleagues [51] and Bernard-Gallon and coworkers [52]. From a molecular standpoint, genistein seems to exert pleiotropic effects, being able to activate various DNA damage checkpoints, promoting cell cycle arrest, mitotic catastrophe and cell death (depleting G1 population of cells, leading to an accumulation at G2/M and activating the apoptotic cascades, up-regulating caspase-3 and caspase-7 as well as p38/MAPK and ATM/Chk2/Cdc25C/Cdc2, and down-regulating the Bcl-2-Bax and NF-κB/p65 complexes) [53,54,55]. Furthermore, genistein finely tunes different angiogenetic cascades [56,57].

Kotsopoulos and collaborators [58] designed a case-control study recruiting 48 cases matched with 96 controls, to evaluate the impact of a panel of 14 micronutrients on breast cancer risk in women carrying the BRCA1 mutation. Iron uptake was protective, whilst antimony consumption was a risk factor.

Pirouzpanah and colleagues [59] assessed the relationship between dietary uptake of one-carbon metabolism-related nutrients and BRCA1 promoter hypermethylation and expression status in dissected breast cancer tissue samples from 146 Iranian women. Dietary folate and cobalamin uptake inversely correlated with methylated BRCA1, with an age-dependent effect of nutrient intake on promoter methylation status.

Dziaman et al. [60] fond that selenium supplementation in adnexectomized women carrying BRCA1 mutations up-regulated the base excision repair (BER) enzymes’ activities (in particular, the activity of hOGG1 glycosylase).

### 3.2. BRCA2, Diet and Breast Cancer

The BRCA2 protein is encoded by a gene located to 13q12.3. Mutated BRCA2 can lead to a variety of disorders, including Fanconi anemia, breast, ovarian, prostate and pancreatic cancer, and malignant melanoma, among others [61,62].

Similar to BRCA1, genistein supplementation was found to up-regulate BRCA2 levels [52]. Vissac-Sabatier and coworkers [63] demonstrated that genistein but not daidzein up-regulated BRCA2.

### 3.3. BRCA1/BRCA2, Diet and Breast Cancer

The BRCA status was found to correlate with vegetable consumption and fruit uptake (OR 0.27 (95% CI 0.10–0.80)) [64,65].

The “Korean Hereditary Breast Cancer Study” showed that soy intake was associated with a lower risk of breast cancer in BRCA mutation carriers (especially BRCA2 mutations). Meat intake was not associated, instead, with BRCA status [66].

Furthermore, Bissonauth and collegues [67] performed a nested case-control study within a cohort of 5660 French-Canadian women (280 of which were affected by breast cancer and nongene carriers of mutated BRCA gene, whereas the remaining 280 were unaffected and nongene carriers). High energy intake, coffee and alcohol consumption correlated with an increased risk of breast cancer.

Caëtano and coworkers [68] studied the effects of soy isoflavones (daidzein and genistein) on human breast cancer cells in vitro. Authors found an over-expression of various genes belonging to the BRCA1 and BRCA2 pathways.

### 3.4. COMT, Diet and Breast Cancer

Catechol-O-methyltransferase (COMT) is an estrogen-metabolizing enzyme catalyzing the O-methylation of catechol estrogens, utilizing S-adenosylmethionine (SAM) as a source of methyls. Some of its haplotypes have been associated with an increased risk of developing breast cancer [69] and mammographic density [70]. In particular, the val108met COMT polymorphism, which results in a 3-4-fold decrease in activity, is associated with increased breast cancer risk [71].

Folate, the uptake levels of which have also been demonstrated to correlate with breast cancer risk, and other micronutrients belonging to the folate metabolic pathway, can affect the concentrations of SAM and S-adenosylhomocysteine (SAH) [72].

Among women carrying at least one low-activity COMT allele, those consuming tea displayed a significantly decreased risk of developing breast cancer (OR 0.48 (95% CI 0.29–0.77)) with respect to non-tea drinkers [73]. The randomized, controlled “Minnesota Green Tea Trial” (MGTT) was recently designed and carried out in order to further shed light on this issue [74].

### 3.5. Vitamin D, Diet and Breast Cancer

Epidemiological surveys have found an inverse association between vitamin D, calcium intake and mammographic breast density [75,76], as well as an inverse association between high sunlight exposure and breast cancer risk [77]. These associations may be stronger for premenopausal than postmenopausal women due to interactions between vitamin D, the vitamin D receptor (VDR), estrogen and insulin-like growth factor-I (IGF-I) [78].

Various VDR polymorphisms exist: Cdx-2, FokI, BsmI, ApaI, and TaqI. FokI polymorphism was related to an increased risk for breast cancer, whereas the BsmI polymorphism conferred a decreased risk [79,80].

Calcitriol (1,25(OH)_2_D) is involved in several biological functions, including inhibiting cell proliferation, inducing differentiation and apoptosis, and inhibiting angiogenesis in normal and breast cancer cells [81,82,83], suppressing high-fat diet-induced mammary tumorigenesis in a variety of animal models, like rats.

Knight and collaborators [84] performed a case-control study, recruiting a sample of 972 women with newly diagnosed breast cancer versus 1135 healthy controls. Reduced breast cancer risk was associated with increased sun exposure in the age-group 10–19 years, cod liver oil use and milk uptake. Vitamin D-related exposures, outdoor activities, use of sunscreen, dietary contributions were protective factors, as corroborated by further studies.

More frequent sun exposure during adolescence was associated with a reduction up to 35% in breast cancer risk later in life, although this can only be observed in women with light constitutive skin pigmentation and not in subjects with medium or dark pigmentation [85].

The effect of sun exposure could be age-dependent, with a milder protection seen for people aged 20–29 years, whilst no protection can be observed for people over age 45.

Epidemiological studies conducted in countries with different latitudes (for instance, Norway, Japan) showed a correlation between different annual UV exposure levels and breast cancer risk. Furthermore, prognosis is slightly better (15%–25%) for women diagnosed and/or treated in the summer versus winter [86,87].

### 3.6. 5-LO, ALOX5AP, Diet and Breast Cancer

5-Lipoxygenase (5-LO) is an enzyme which catalyzes the first two steps of the biosynthesis of leukotrienes from arachidonic acid [88].

A statistically significant relation between the ALOX5AP (5-lipoxygenase-activating protein gene) −4900 A > G polymorphism and dietary linoleic acid uptake was found. Among women consuming a diet high in linoleic acid (consumption greater than 17.4 g/day), carrying the AA genotype was associated with higher breast cancer risk with respect to women carrying other genotypes [89].

### 3.7. CYP17, Diet and Breast Cancer

CYP17 is a major enzyme involved in human steroidogenesis [90]. CYP17A1 (also known as P450c17 or P450sccII) catalyzes the formation of all endogenous androgens [91].

Among premenopausal women, the risk of developing breast tumor did not correlate with genetic status. A statistically significant modifying effect of genotype on plasma enterolactone-related tumor risk could be reported, with plasma enterolactone significantly inversely being correlated with the risk of developing breast cancer in women carrying the A2A2 genotype. With respect to women carrying the A1A1 genotype and with the lowest supply of enterolactone, the decrease in risk associated with high enterolactone concentrations was paralleled by a reduced risk of breast tumor regardless of the genotype. Concerning genistein, no clear-cut evidence for a differential effect based on the CYP17 genotype could be detected [92].

### 3.8. Aromatase (CYP19), Diet and Breast Cancer

Aromatase (CYP19A1), an enzyme belonging to the family of P450 cytochromes (P450arom) with a crucial role in the biosynthesis of estrogens (C18 steroids) from androgens (C19 steroids), is encoded by the CYP19 gene localized to 15q21.1 [93].

Kopp and collaborators [94] performed a case-control nested study within the Danish “Diet, Cancer and Health” cohort (687 cases and 687 controls). Authors found significant interactions between alcohol intake, CYP19A1 polymorphisms and hormone levels: homozygous variant allele carriers displayed an increased risk of breast tumor.

### 3.9. NAT1/NAT2, Diet and Breast Cancer

Some polymorphism of arylamine N-acetyltransferases types 1 and 2 (NAT1, NAT2) confer a higher risk of developing breast cancer. Furthermore, they correlate with disease progression [95,96,97,98].

According to Egeberg and collaborators [97], with respect to slow acetylators, the incidence ratio of breast cancer among those carrying the fast NAT1 genotype was 1.43 (95% CI 1.03–1.99) and among those with the intermediate/fast NAT2 allele, risk was 1.13 (95% CI 0.83–1.54). Interaction analyses could demonstrate that the statistically significant relationships between meat (specifically, read meat) consumption and the risk of developing breast tumor were limited to women carrying the intermediate/fast NAT2 genotype.

### 3.10. MTHFR/TYMS, Diet and Breast Cancer

Some polymorphisms of MTHFR, an enzyme catalyzing the conversion from 5,10-methylenetetrahydrofolate to 5-methyltetrahydrofolate, confer a higher risk of developing breast cancer [99].

In women with a daily folate uptake less than 133.4 μg, a weekly or daily green tea uptake inversely correlated with breast tumor risk with respect to less green tea consumption (OR 0.45 (95% CI 0.26–0.79)]. Among women with high folate intake, green tea consumption was not found to be associated with breast cancer. A similar trend was reported for those women carrying the high-activity MTHFR/TYMS genotypes and frequently (weekly or daily) consuming green tea (OR 0.66 (95% CI 0.45–0.98)). This association was even stronger among women with low folate uptake but failed to achieve the significance threshold among those carrying the low-activity genotypes [100]. 

Folate uptake from diet was associated with a higher breast cancer risk in women carrying the MTHFR 677CT/TT-1298AA mutation, but an inverse association was described in compound heterozygous women. A statistically significant interaction was noted between dietary folate intake and the C allele. The T allele correlated with increased breast cancer risk in women aged 55 years and older (adjusted OR 1.34; 95% CI (1.01–1.76)). Homozygosis for the C allele conferred a higher risk in women aged 45–55 years (adjusted OR 1.89 (95% CI 1.09–3.29)) [101].

A 62% increased risk of breast tumor could be observed in a sample of postmenopausal women carrying the TT genotype. Women with a higher number of variant T alleles had a higher risk of developing breast cancer. A modifying effect due to the uptake of B vitamins was described. The most relevant MTHFR-breast cancer risks were reported among women consuming low amounts of folate and vitamin B6, whereas no statistically significant increased risks could be found among women with higher uptakes [102].

### 3.11. Estrogen Receptors, ER-Alpha and ER-Beta, Diet and Breast Cancer

ER-α plays a major role in different steps (initiation and proliferation) of breast cancer, in that it up-regulates the expression of oncoproteins, whereas it down-regulated the levels of cell cycle inhibitors, such as P21 [103,104].

Dietary green tea polyphenol, (-)-epigallocatechin-3-gallate (EGCG), can restore ER-α expression in an in-vitro model and can also result in a remodeling of the chromatin structure of the ER-α promoter by epigenetic changes, including altered histone acetylation and methylation status, as well as in a decreased binding of the transcription repressor complex at the level of the regulatory region of the ER-α promoter [105]. 

Similar results were obtained by Donovan and collaborators [50], who showed that genistein supplementation can result in the restoration of an ER-α-mediated response, thus imparting the sensitivity of triple-negative breast cancer to anti-estrogen therapy.

### 3.12. Oxidative Stress Pathway, MPO, CAT, HO and NOS, Diet and Breast Cancer

Myeloperoxidase (MPO), catalase (CAT), heme oxygenase (HO) and nitric oxid synthase (NOS) encode proteins with a prominent role in the oxidative stress network. HO mediates the response to several drugs, including pharmorubicin and piperlongumine [106,107,108,109], whereas CAT plays a major role in chemoresistance [110]. NOS has emerged in the last years as a potentially druggable target in the therapy of breast cancer [111].

The study by Li and colleagues found that women consuming low amounts of fruits and vegetables and carrying low-risk alleles displayed a higher breast cancer risk. These results, taken together, seem to suggest the role of antioxidants (either endogenous or exogenous) in the process of breast tumorigenesis [112].

### 3.13. EZH2, Diet and Breast Cancer

The “polycomb group (PcG) protein, enhancer of zeste homologue 2” (EZH2), is over-expressed in breast tumor, and has been correlated with cancer growth, proliferation and metastasis [113,114].

The consumption of dietary omega-3 polyunsaturated fatty acids (PUFAs) can finely tune and modulate the levels of EZH2 in breast cancer cells. The supplementation with omega-3 PUFAs, but not omega-6 PUFAs, can result in down-regulation of EZH2, and upregulation of E-cadherin and insulin-like growth factor binding protein 3 (IGF-BP3) [115].

### 3.14. SHBG, Diet and Breast Cancer

Sex hormone binding globulin (SHBG) is a major biomarker of breast cancer risk, diagnosis and prognosis [116,117].

For the rs6259 SHBG polymorphism, an inverse association was found in postmenopausal Japanese women carrying the GG genotype (OR for the highest versus the lowest tertiles of 0.50 (95% CI 0.29–0.87), P_for trend_ < 0.01), and in non-Japanese Brazilian subjects carrying at least one A allele (OR for consumers versus non-consumers of 0.21 (95% CI 0.06–0.77)) [118].

### 3.15. RASSF1A, Diet and Breast Cancer

“RAS-association domain family 1 isoform A” (RASSF1A) methylation is an important biomarker of breast tumor. According to a review of the literature and meta-analysis, the epigenetic modification of the gene displays a sensitivity and a specificity of 0.49 and 0.95, whilst the diagnostic odds ratio (DOR) and the area under the curve (AUC) resulted in 19.0 and 0.83, respectively [119].

Whereas Pirouzpanah and colleagues [58] found a negligible effect of nutritional epigenomics on RASSF1A status, utilizing an animal model, Rodríguez-Miguel and colleagues [120] investigated the effects of oil-enriched diets resulting in hypermethylation of the RASSF1A promoter region.

### 3.16. RARB, Diet and Breast Cancer

Retinoic acid receptor beta (RARB) encodes for a nuclear receptor.

Pirouzpanah and collaborators [59] found that high dietary intake of riboflavin and pyridoxine contributes to highly methylated promoters, thus reducing breast cancer risk.

### 3.17. GSTP1, Diet and Breast Cancer

“Glutathione S-transferase P type 1” (GSTP1), an enzyme encoded by the GSTP1 gene, is responsible for the xenobiotic metabolism [121,122]. Recently, it has emerged as a potentially druggable target, especially in triple-negative breast cancers. Its inhibitor, piperlongumine, appears to be promising.

Lee and collaborators [123] recruited 3035 cases matched with 3037 population controls sampling from the “Shanghai Breast Cancer Study” A GSTP1 haplotype (namely, the Val/Val genotype) significantly correlated with a higher risk of developing breast tumor (displaying an OR of 1.50 (95% CI 1.12–1.99)). Women carrying the mutation and consuming low amounts of cruciferous vegetables had a higher risk than the women carrying other mutations. However, the effects of the interaction between the consumption of low amounts of cruciferous vegetables and the Val/Val genotype seemed to be age-dependent, being detected mainly in premenopausal women.

### 3.18. Genes, Diet and Breast Cancer

The main findings of the interactions between gene polymorphisms and nutrients and their effects in terms of breast cancer risk are summarized in Table 2.

## 4. Conclusion

Breast cancer imposes a tremendous burden in terms of epidemiological, societal implications and economic costs. The association between breast cancer and diet is complex, multi-factorial and non-linear. Classical nutritional epidemiological surveys have demonstrated conflicting results, showing a modest correlation between diet and breast cancer risk (apart from alcohol). We can speculate that this may be due to the complexity of breast cancer, which is a multi-faceted, highly heterogeneous disorder. Histological and, recently, molecular classifications have contributed to underpin a rather complex picture.

Therefore, we need molecular details: we need nutrigenetics and nutrigenomics. Nutrigenomics and allied disciplines can foster advancements in knowledge in this field, shedding light on the molecular background of breast cancer tumorigenesis, and paving the way towards personalized treatments [124,125,126,127].

## Figures and Tables

**Table 1:** An overview of the effects of major nutrients in terms of breast cancer risk.

Nutrient	Main Findings	Reference
Alcohol	Higher risk	[35,36]
	Different effects according to menopausal status	[37]
	Different effects on survival	[38]
Coffee	No clear effect	[39,40,41,42]
Dietary products	Dose-dependent effect	[18]
Egg	Higher risk	[19,20]
Fat	Higher risk	[13,14]
Fish	Different effects based on the fatty acids assessed	[31,32,33,34]
Folate	Dose-dependent effect	[21]
Fruit	Different effects based on the type of fruit and study design	[22,23,24,25,26]
Meat	No clear effect	[15,16,17]
Mediterranean diet	No clear effect	[8,9,10,11,12]
Soy	Different effects based on ethnicity	[29,30]
Tea	No clear effect	[43,44]
Vegetables	No clear effect	[22,27,28]

**Table 2:** An overview of the main interactions between gene polymorphisms and nutrients and their effects in terms of breast cancer risk.

Breast Cancer-Related Gene	Interaction between Gene Polymorphism and Nutrient	Effect of the Interaction	Reference
BRCA1	Coffee consumption and BRCA1 mutation	Lower risk	[49]
	Genistein intake and BRCA1 mutation	Lower risk	[50,51,52]
	Selenium supplementation and BRCA1 mutation	Lower risk	[60]
	Folate and cobalamin intake and BRCA1 promoter methylation status	Lower risk	[59]
	Iron consumption and BRCA1	Higher risk	[58]
	Antimony consumption and BRCA1	Higher risk	[58]
	Vegetables and fruits consumption and BRCA1	Lower risk	[64,65]
BRCA2	Soy intake and BRCA2 mutation	Lower risk	[66]
	Genistein and BRCA2	Lower risk	[52,63]
	Vegetables and fruits consumption and BRCA2	Lower risk	[64,65]
CAT	Low-risk allele and low fruits and vegetables consumption	Higher risk	[112]
COMT	Low-activity COMT variant and green tea consumption	Lower risk	[73,74]
CYP17	Genistein uptake and CYP17 genotype	No clear effect	[92]
CYP19A1	Homozygous variant allele and alcohol consumption	Higher risk	[94]
ER-α	Green tea polyphenol, (-)-epigallocatechin-3-gallate, and genistein epigenetically modify chromatin structure of the gene	Lower risk	[50,105]
EZH2	Omega-3 PUFAs and EZH2	Lower risk	
GSTP1	Val/Val genotype X low consumption of cruciferous vegetables	Higher risk (in premenopausal women)	[123]
HO	Low-risk allele and low fruits and vegetables consumption	Higher risk	[112]
5-LO/ ALOX5AP	−4900 A > G polymorphism and dietary linoleic acid uptake	Higher risk	[89]
MPO	Low-risk allele and low fruits and vegetables consumption	Higher risk	[112]
MTHFR/TYMS	MTHFR 677CT/TT-1298AA mutation and folate uptake	Higher risk	[101]
	Green tea uptake and MTHFR genotype	Lower risk	[100]
	Low amount of vitamin B6, low folate intake and MTHFR genotype	Higher risk	[102]
NAT1/NAT2	Intermediate/fast NAT2 genotype and red meat consumption	Higher risk	[97,98]
NOS	Low-risk allele and low fruits and vegetables consumption	Higher risk	[112]
RARB	High dietary consumption of riboflavin and pyridoxine contribute to highly methylated promoters	Lower risk	[59]
RASSF1A	Different nutrients, including oil-enriched diets	No clear effect	[59,120]
VDR	Milk uptake and VDR polymorphisms	Lower risk	[84]
